# Oxytocin as Treatment for Social Cognition, Not There Yet

**DOI:** 10.3389/fpsyt.2019.00930

**Published:** 2020-01-09

**Authors:** Amaia M. Erdozain, Olga Peñagarikano

**Affiliations:** ^1^ Department of Pharmacology, University of the Basque Country UPV/EHU, Leioa, Spain; ^2^ Centro de Investigación Biomédica en Red en Salud Mental CIBERSAM, Leioa, Spain

**Keywords:** oxytocin, social cognition, clinical trial, autism, schizophrenia, depression

## Abstract

In a short time, oxytocin has progressed from being a regular hormone involved in parturition and breastfeeding to be possibly the neuromodulator that has gathered the most attention. Attributed many positive roles in the modulation of different aspects of social behavior, such as bonding, empathy, cooperation, trust, and generosity, as well as roles as a natural anxiolytic and antidepressant, the expectations on oxytocin becoming a treatment for a number of disorders with associated social deficits have dramatically raised over the last years. However, despite the field has been investigating oxytocin’s role in social behavior for over twenty years, there are still many unknowns on oxytocin’s mechanisms of action and efficiency and the increasing number of clinical trials administering oxytocin to different clinical groups seem to disagree in its properties and report in most cases conflicting results. This has led to some disappointment among researchers and clinicians as oxytocin might not be the miraculous molecule that works in a “one size fits all” fashion initially considered. Conversely, this down-side of oxytocin might merely reflect the complexity of its neurotransmission system. The current reality is that, although oxytocin seems to have potential therapeutic value, there are key questions that remain unanswered as to decide the optimal target groups and treatment course. Here, we present an overview on critical points regarding the oxytocin system in health and disease that need to be better understood to establish its therapeutic properties and to decide who could benefit the most from its treatment.

## Introduction

The field of clinical behavioral neuroscience has rarely experienced such an enthusiasm for a single molecule as the one elicited by oxytocin (OXT). Over the past decade, the peptide hormone that regulates birth-related processes has been transformed into a somewhat magical neurotransmitter that modulates an individual’s social abilities. The rapidly growing field of OXT research has attributed this molecule miraculous properties as enforcer of trust, empathy, generosity, altruism, or even love, to an extent that commercially available nasal sprays are advertised to enhance an individual’s social skills. Despite many doubtful reports endorsing OXT’s power as a miracle potion, rigorous, and sound research has indeed proven the role of OXT in regulating social cognition (1–3). Thus, in the recent years, there has been an explosion in the number of clinical trials to test OXT’s efficiency in improving social deficits in a variety of conditions with associated social dysfunction including both neuropsychiatric disorders such as autism, schizophrenia, depression, social anxiety, drug addiction, as well as neurodegeneration-related disorders ranging from dementia and Huntington’s disease to ageing. Although the potential success of OXT as a pharmacotherapy is exciting, the increasing number of treatment studies report conflicting results and several issues need to be addressed before accepting the effectiveness of this molecule. Important questions such as selection of the clinical sample, dosage, timing, effect by sex, behavioral outcome measure, neurobiological effect or correlation with OXT-related biomarkers, to potentially identify best responders, have not been systematically addressed. In the present review, first we summarize important aspects of the OXT signaling system which should be considered to inform treatments; later, we present evidence linking alterations in the OXT system in neuropsychiatric disorders characterized by social dysfunction and last, we summarize results of OXT treatments in clinical trials together with an overview on critical points that need to be better understood to establish its therapeutic properties and to decide who could benefit the most from its treatment.

## The Oxytocin System

Although OXT is synthesized to some extent in certain peripheral areas, the majority is produced in neurons located in the paraventricular (PVN) and supraoptic (SON) nuclei of the brain hypothalamus. It is released both to the bloodstream to modulate several peripheral functions, and within the brain, where it acts as a neuromodulator. OXT-producing neurons project to various brain areas including limbic regions, brain cortex, and brainstem (4), where they release OXT in an action potential-dependent manner. In addition, OXT, like other neuropeptides, can be released independently of action potentials by exocytosis through the whole neuron, diffusing in the extracellular space (5). An important molecular modulator of this type of release is the transmembrane protein CD38 (cluster of differentiation 38). OXT’s local release is thought to be responsible for its well-known self-priming process, in which OXT stimulates its own release in a positive feedback loop, presumably through activation of its own somato-dendritic autoreceptor (6). This is important for treatment design, as despite the short half-life reported for the peptide in brain (about 20 min), a single dose of OXT has been shown to have a behavioral effect that lasts for several hours (6). In addition to its own receptor, OXT expressing neurons also express a variety of other receptors that, when activated, can trigger OXT release. The most studied is the melanocortin 4 receptor (MC4R), and current efforts are made to develop pharmacotherapies that induce endogenous OXT release, to achieve physiological-like conditions.

The oxytocin receptor (OXTR) belongs to the G-protein-coupled seven transmembrane receptor superfamily and is located throughout the brain both in neurons and glia (7, 8). OXTR can be coupled to Gαq and Gαi/o, activating or inhibiting different intracellular signaling cascades [reviewed recently (9)]. An interesting hypothesis is that these different pathways are engaged depending on the local concentration reached by OXT. Thus, low concentrations activate Gαq whereas higher concentrations promote the activation of Gαi/o proteins (10). Further, upon continuous agonist exposure OXTR undergoes rapid and extensive internalization and desensitization, through the classical clathrin-mediated pathway (11). Such complex regulation of the OXTR signaling pathway indicates the need of dose-response studies that could inform treatment design.

Further, the existence of a critical period when OXT modulation might influence developmental processes achieving long-lasting effects, has been suggested. Seminal studies in voles have shown that early OXT administration has long-term consequences in both OXT expression in the PVN (12) and social behavior in adults (13). In fact, in rodents, the OXT system undergoes specific changes from birth throughout the lifespan: the number and complexity of OXT projecting neurons gradually increases postnatally, reaching maturity at juvenile age and the expression of OXTR also shows a specific developmental pattern (4). This precise developmental regulation of the OXT system is thought to modulate different aspects of brain development. Interestingly, in humans, a peak in OXTR binding has been observed in the ventral pallidum during early childhood in typically developing individuals, but this peak was absent in children with ASD (14). Determining whether these changes can be permanently reversed or compensated through early manipulations is still unkown.

## The Oxytocin System in Psychiatric Disorders of Social Cognition

As described above, for a functional OXT system, proper production and release of OXT, together with a precise expression of OXTR is needed. Once the role of the OXT system in modulating affiliative behavior across vertebrate species was established, efforts in translating these findings to the clinic begun (15). Special emphasis has been placed on trying to identify biomarkers for disorders with altered social cognition. The most studied players within the OXT system are the OXT peptide, the OXTR and CD38, since both OXT (its synthesis, processing, storage, and release), as well as its receptor, could be affected in a pathological state. Identifying biomarkers would not only help diagnosis, but also serve as a stratifying factor to ascertain which patients could benefit the most from OXT’s treatment.

### Peripheral OXT Measurements

At a biochemical level, there is still much debate on whether peripheral OXT levels (in blood, urine, or saliva) are correlated with OXT levels in brain (16). Nevertheless, studies measuring OXT concentrations in plasma in psychiatric populations show contradictory results, reporting in some cases no differences, and in others both lower as well as higher OXT levels in patients compared to controls. For example, some studies found lower plasma OXT associated with autism (17, 18), depression (19), and schizophrenia (20, 21) while other studies described higher OXT levels associated with autism (22, 23) and depression (24, 25). There are several factors that might explain some of these contradicting results. First, current methodology used to measure OXT has not been rigorously validated. Traditional immuno-assays (such as ELISA or RIA) require a sample extraction process to eliminate potentially interfering proteins that at the same time eliminates protein-bound OXT (26). Newer methods using liquid chromatography/mass spectrometry (LC/MS) separate first OXT from any bound protein, measuring total (free plus protein-bound) OXT, although the functional significance of free vs. bound OXT remains unknown (26). Thus, development and/or validation of straight-forward, sensitive, and specific assays for OXT quantification is needed. In this case, accurate measurements of OXT release into both blood and brain could be systematically monitorized and the question of whether peripheral and central OXT levels are linked, and are correlated to a given behavioral phenotype, could finally have a response.

### Genetic and Postmortem Studies

Genetic studies provided converging evidence that variation in the form of single nucleotide polymorphisms (SNP) in genes within the OXT signaling system confer risk to psychiatric endophenotypes related to social dysfunction. Special attention has been drawn to the OXTR, since seminal studies performed in voles showed that the amount of parental care, affiliative bonding, and even aggression toward conspecifics, seems to be modulated by differences in the levels of OXTR expression and/or its differential distribution throughout the brain (27). In fact, in voles, genetic variation within the OXTR gene has been shown to affect OXTR density in a region-specific manner. Specifically, a certain single SNP in the OXTR gene modulated OXTR expression in the nucleus accumbens, a structure widely implicated in the processing of reward, and correlated with the propensity of the animals to form social attachments (28). However, whether this is also true for the identified human variants is uncertain, and the effect of most identified SNPs on gene expression or protein function remains to be elucidated. It is noteworthy that studies investigating the expression of the OXTR (through qPCR or receptor binding autoradiography) in postmortem brain tissue of neuropsychiatric disorders report, in most cases, a different distribution between cases and controls. For example, in individuals with autism, OXTR binding has been found to be increased in cortical and decreased in subcortical structures (14). Increased OXTR expression has been reported in the prefrontal cortex of individuals suffering from major depression (29) and alcohol use disorder (30, 31). Last, decreased OXTR mRNA was found in several brain areas of subjects with schizophrenia (32). In search for the causal factor of such differences, recent data highlight the role of DNA methylation on the transcription levels of OXTR. In fact, in mice, specific CpG sites in the promoter of the OXTR gene have been shown to be differentially methylated between brain regions expressing different levels of OXTR (33). In humans, hypermethylation of the OXTR gene was found to be associated with decreased levels of its mRNA in the temporal cortex of individuals with ASD (34). In addition to OXTR expression, few studies have investigated levels of the OXT peptide expression within the brain. As an example, subjects with major depression showed increased number of OXT-immunoreactive neurons in the PVN of the hypothalamus (35). Current research aims at ascertaining the endogenous, pharmacological, and environmental factors regulating OXT/OXTR expression.

## Oxytocin in Clinical Trials

OXT administration has been tested to improve social cognition in a variety of clinical groups, including autism, depression, schizophrenia, drug addiction, and neurodegenerative disorders. However, clinical trials have yielded inconclusive results, with some reports supporting a significant positive effect and others suggesting a null effect. Several meta-analyses have been performed to address this issue (summarized below), which albeit still inconsistent, highlight the differences that might account for this inconsistency, such as the clinical sample studied, which differ in the social behavioral domain affected, and the specific outcome of social cognition being evaluated (see below section “oxytocin treatments, things to consider”).

One of the main limitations of conjunctly analyzing the data in a meta-analysis is in fact the huge variability in the outcome measure used in each study. Regardless, most studies include affective tests, such as emotion recognition and expression of emotions, as well as theory of mind as the main social behavior measures. Still, meta-analyses focusing on these common parameters show contradicting results. For example, Keech et al. co-analyzed 17 studies including individuals with autism, schizophrenia, and Prader-Willi syndrome and found no effect on emotion recognition, a trend in emotion expression, and a significant effect on theory of mind (36). However, another study that included 15 reports on a different clinical population (i.e. autism, borderline personality disorder, depression, schizophrenia, and drug dependence), found that OXT did not significantly influence any of the same behavioral outcomes (37). These differences might be due to the specific clinical populations included in each work, as another study covering 19 clinical trials including individuals with autism, social anxiety, postnatal depression, obsessive-compulsive disorder, schizophrenia, borderline personality disorder, and post-traumatic stress, showed that only the autism group presented a significant effect on social parameters (i.e. emotion recognition) (38), indicating that studies focused on a specific diagnosis, with a common behavioral domain affected, could be helpful. In fact, in a quantitative meta-analysis of 16 fMRI studies of i.n. OXT treatments in autism, borderline personality disorder, depression, social anxiety, and post-traumatic stress disorder, OXT was found to have diagnosis-specific neurobiological effects, increasing dorsal anterior cingulate activity in ASD and decreasing amygdala activity in social anxiety disorder (38). Still, a meta-analysis focusing specifically on ASD found no significant behavioral effect of OXT in neither the social nor the repetitive behavior domain (39). With respect to studies focusing specifically on schizophrenia, it is important to mention that the great majority of clinical trials have focused on positive (delusions and hallucinations) and/or negative (diminished motivation, social functioning, and ability to express emotions) symptoms as a whole, and few studies evaluated specifically social cognition outcomes. In general, no improvement of negative symptoms in schizophrenic patients has been reported (40). However, when focusing specifically on social cognition, a significant effect in outcomes for high-level social cognition (such as mentalizing and theory of mind) has been found (41). In borderline personality disorder, OXT had a beneficial impact on recognition and discrimination of emotions and on hypervigilance toward social threats, whereas it could hinder trust and cooperation (42). Recent studies are currently examining OXT’s potential beneficial effects on social cognition in conditions other than psychiatric disorders. In fact, if we assume that OXT modulates certain aspects of social cognition, it is reasonable to think that it does so regardless of diagnosis and/or condition. Thus, OXT could potentially improve the processing of emotional faces in neurodegenerative diseases such as Huntington’s disease (43) and frontotemporal dementia (44, 45). Further, OXT is currently being tested as treatment of social cognitive difficulties shown by healthy ageing individuals (46), where OXT does seem to improve certain aspects of social cognition (i.e. theory of mind), although not specifically in ageing population. Additional studies stratifying cases by the affected behavioral domain, rather than diagnosis per se, and with a common behavioral outcome are needed to ascertain OXT’s role in improving certain domains of social behavior (see below).

## Oxytocin Treatments, Things to Consider

As noted from the studies above, the effectiveness of OXT in improving social cognition is still at debate. Several issues such as selection of the clinical sample, dosage, timing, effect by sex and/or age, and the choice of behavioral outcome measure could be responsible for contrasting results among trials. Further, the associated neurobiological effect or correlation with OXT-related biomarkers, to potentially identify best responders, have not been systematically addressed. Below we present the main gaps that need to be filled in order to finally attribute OXT its potentially therapeutic effect.

### Patients’ Sample

Although the number of human studies testing OXT’s efficacy in several disorders keeps increasing, the sample size is in most cases small and studies are underpowered. The outcome measures are also very diverse, which limits the possibility of conjunctly analyzing the data in a meta-analysis, as described above. Also, the clinical phenotype of the patients must converge in the affected social-behavioral domain, since even in the case of a common diagnosis, the clinical heterogeneity among the affected individuals is usually big. It might be worth focusing on an affected behavioral domain, rather than diagnosis. Also, the general status of the patients must allow for improvements in the behavioral outcome to be measured, ensuring that the presence of associated symptoms does not obscure interpretations of social cognition. In addition, the potential different effects based on age and sex are usually not considered. Female subjects may respond differently to OXT treatments because of potential differences in basal levels of OXT or differential expression or distribution of OXTR, since sex hormones are known to modulate the OXT system. Specifically, estrogen has been shown to regulate the expression of OXTR in brain (47). Last, the mainly postnatal development of the OXT system, as described above (48), suggests that during this early-life temporal window the OXT system might be more susceptible for long-lasting physiological and behavioral effects.

### Behavioral Outcome

A primary issue in designing randomized clinical trials is to establish the outcome measure that defines improvement in impaired behaviors. As disorders diagnosed by behavioral evaluation, performance in a certain behavioral task affected in the disorder, a clinical interview or questionnaire, or even self-report are usually the parameters measuring treatment effectiveness. Albeit some common behavioral measurements are used, such as specific affective tests, the published outcome measures for OXT trials are, in general, very variable, with numerous non-standardized assessments. Thus, OXT has been shown to improve social cognition in many different ways, such as comprehension of affective speech in ASD (49) and facial emotion recognition in depression (50), where participants have to infer emotion from read-aloud sentences or pictures of faces, respectively. Understanding of indirect cues and recognition of social faux pas (i.e. a behavior not socially accepted) have been reported to be improved by OXT in schizophrenia (51), and improved eye contact during a social challenge has been reported in social anxiety (52). Attempts at developing standardized protocols to score improvements in social behavior over the course of a randomized clinical trial are needed.

### Dosing

Most trials performed to date show a great inconsistency in their treatment course, with varying doses ranging from 8 to 50 IU, which could explain the discrepancy in some of the results, particularly if different OXTR signaling pathways could be activated depending on the dose, as described above (10). Even if 24 IU has been the dose most studied to date, a much lower OXT dose of 8 IU has also been found to influence social cognition (53). Therefore, it is clear that the effects of i.n. OXT need proper dose-response studies in order to find the optimal effective dose on biology (see below) and behavior. A recent OXT acute dose/response (8–84 IU) study in schizophrenia using neurophysiological (EEG) measurements of social processing has suggested that OXT would produce biological effects in an inverted U-shaped manner, where only mid-range doses produce an effect (54). Besides, the majority of clinical trials administered an acute, single-dose OXT, and there are very limited studies investigating the effects of chronic OXT administration over a period of several months (55, 56).

### Oxytocin Administration, Crossing the Blood Brain Barrier

Despite animal studies showing that systemically administered OXT shows behavioral and even neurological effects (57, 58), it is still at debate whether peripherally administered OXT crosses the blood-brain barrier in appreciable amounts (59). I.n. OXT administration has been proposed as an alternative pathway to bypass this penetrance issue (60). Still peripherally administered OXT might indirectly, through afferent feedback signals from the periphery, stimulate endogenous release within the brain (61). Studies measuring OXT cerebro-spinal fluid (CSF) levels after i.n. administration have generally shown an increase, but with some contrasting results in terms of the temporal profile. In humans, elevated CSF and blood OXT concentrations upon i.n. administration have been reported, with a relatively modest and slower increase in CSF and a much greater and more rapid increase in plasma (61). Similar results were obtained in macaques, but in this study the increase in CSF was detected more rapidly than in the previous one (62). Interestingly, another study in macaques also detected elevated OXT CSF concentrations after i.n. aerosolized administration, but not after i.n. spray or intravenous administration (63). Comparable findings were also observed in mouse and rat brain microdialysates, identifying differential temporal profile after i.n. or intraperitoneal administration (64). The criticism that some authors have raised against these results is that only modest increases in CSF levels are observed, with approximately 0.05% of intranasally administered OXT reaching the CSF within 1 hour (59). Recent studies using labeled molecules add evidence in favor of OXT’s penetrance into the brain. A study in macaques using a novel sensitive and specific quantitative mass spectrometry assay that distinguishes labeled (deuterated) from endogenous OXT, demonstrated CSF penetrance of exogenous deuterated OXT after both i.n. and intraventricular administration. Besides, this report also suggests that peripheral administration does not stimulate central release of endogenous OXT, as its level was not changed (65). Furthermore, using a novel peptide tracer for PET imaging of OXTR, the direct nose-to-brain uptake *in vivo* has also been recently demonstrated in rodents (66). Still, even if we accept that i.n. administered OXT reaches the brain, as animal studies seem to indicate, in what concentration, or whether the effect is due to a concomitant increase in peripheral OXT is not known. Further data on this regard would greatly inform dosing studies.

### Safety

Peripheral supraphysiological OXT levels that may be achieved due to exogenous administration could produce undesirable responses and secondary central effects. Indeed, i.n. OXT administration has been shown to produce a marked acute increase in OXT plasma levels that has an elimination half-life of approximately 30 min (67). Several studies have conducted safety measurements in clinical trials supporting OXT's safety. Thus, a daily treatment with 40 IU of i.n. OXT for 4 months in schizophrenia patients did not affect OXT and vasopressin basal plasma levels, nor cardiovascular, body fluids, and food intake parameters (68). Further, several meta-analysis have characterized the side-effect profile of single dose and long-term i.n. OXT and the results support OXT as well tolerated and safe, side-effects not differing between OXT and placebo (69, 70). However, safety should be further evaluated including larger groups, and focusing on sex, age, dosing, and treatment course and also studying putative side-effect that might occur longtime after treatment.

### Biological Effect

Albeit the still unknown detailed pharmacokinetics of i.n. OXT, functional imaging studies in humans strongly support its neurological effect in brain areas involved in emotion and social cognition, as previously mentioned (71, 72). Specifically, a precise activation pattern of interconnected brain regions seems to be the basis for the behavioral effects observed with OXT (71, 73). However, clinical trials usually perform solely a behavioral readout, not always standardized (discussed above), to ascertain the effectiveness of OXT treatment, without any correlation with a biological effect. Very limited studies include functional data (such as fMRI) in combination with performance in social cognition tasks. Understanding the effect of OXT administration on brain function would greatly inform dosing and efficacy of treatment.

### Correlation With OXT Biomarkers

In addition to functional measures, treatment efficacy should be considered in relation to individual differences in their specific molecular signature of the OXT system. As described above, genetic and epigenetic variation in genes within the OXT pathway correlate with endophenotypes related to social cognition. Future trials aimed at deciphering the efficacy of OXT should consider analysis at both the biological and phenotypic level. In this way, we may be able to distinguish between individuals who respond to OXT treatment (OXT responders) and those who do not (OXT non-responders), possibly based on differences in their specific endogenous OXT activity.

## Conclusion

**Figure 1:**
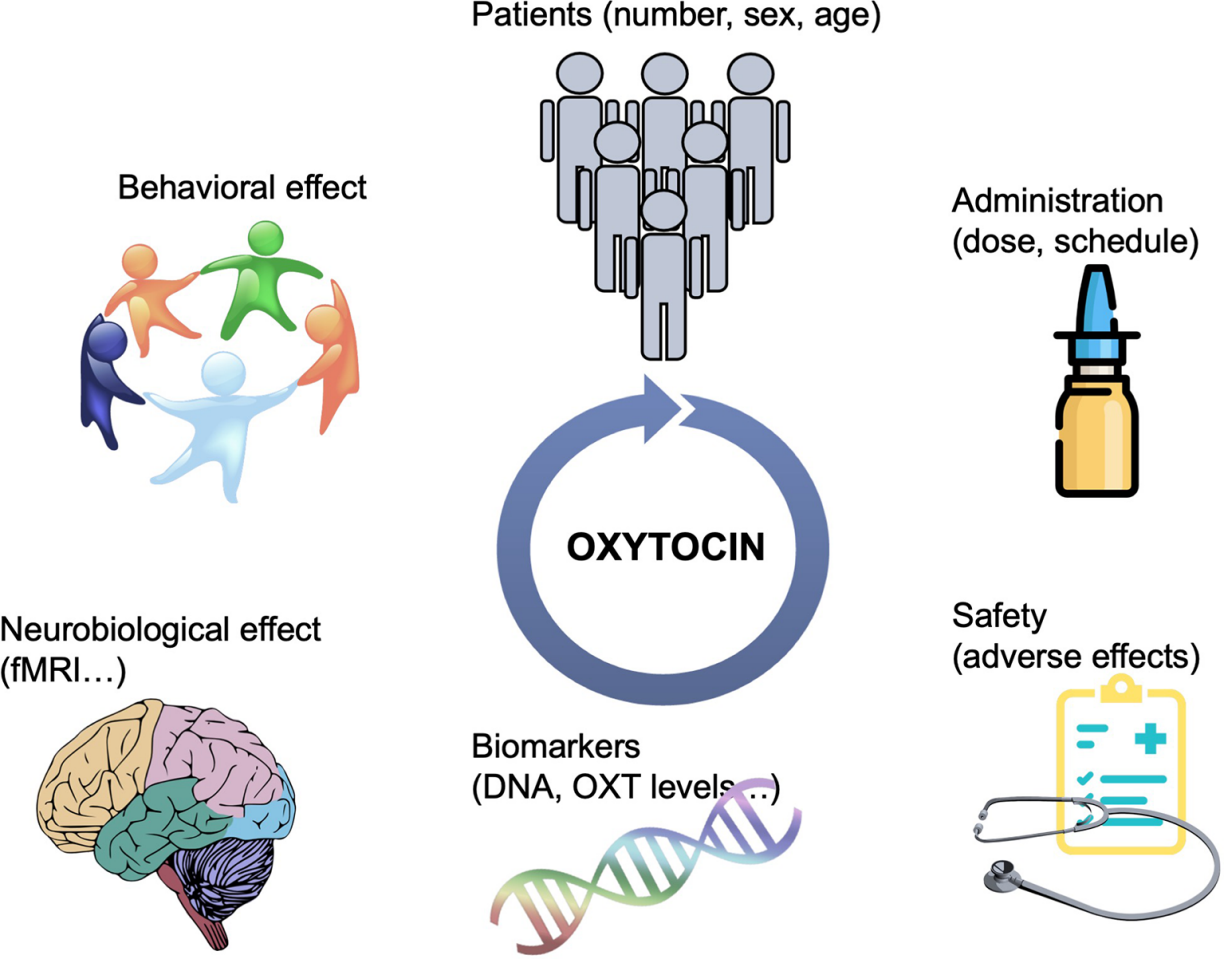
Schematic representation of the strategies needed to guide OXT-based treatments. Bigger sample sizes as well as studies focusing on the potential differential effect of OXT based on sex and age are needed. More efficient delivery paradigms, including the development of specific OXTR modulators would overcame the issue of i.n. administration. The integration of data from multiple aspects, including brain imaging, genetic, and neuroendocrine biomarkers, in conjunction with standardized evaluation of social behaviors, including longitudinal follow-ups, are required to understand the effectiveness of OXT and who could benefit from its treatment.

Oxytocin presents an exciting pharmacological treatment for improving social cognition in a variety of psychiatric disorders. It is easily administered, cost-effective, and with possibly minimal adverse effects. In the recent years, there has been an explosion in the number of clinical trials, mainly in psychiatric populations but also in other conditions affected by social deficits, to test OXT’s efficiency in improving social dysfunction. However, there are still major gaps in this area of research. Although the role of OXT in the modulation of social behavior is today indisputable, the field of OXT-based pharmacotherapy is filled with many small trials that report incongruent results. There are several matters that have not been systematically addressed when designing clinical trials that are critical to inform treatments. The integration of data from imaging, genetic, and neuroendocrine biomarkers, in conjunction with standardized evaluation of social behaviors, including longitudinal follow-ups, are required to understand the effectiveness of OXT and who could benefit from its treatment (**Figure 1**). In addition, there may be alternative means of manipulating the central OXT system to gain efficacy and selectivity, including more efficient i.n. delivery paradigms, or the development of small molecule agonists, positive allosteric modulators or functional selective ligands, activating a specific G protein signaling pathway (10). Endogenous OXT release could also be stimulated pharmacologically to achieve more physiological-like conditions; for instance, MC4R agonists potentiate central OXT release (74) and rescue social deficits in a mouse model of autism (75). In all, OXT has a great potential to ameliorate social dysfunction in a broad range of psychiatric disorders. Efforts should be made to understand its neurobiological mechanism of action and effect based on individual variability in underlying neuropeptide biology.

## Author Contributions

AE and OP contributed equally in drafting the manuscript and adding critical information in the different sections.

## Funding

This work was funded by the Spanish Ministry of Science, Technology and Research, the Spanish State Research Agency and European Regional Development Fund (MCIU/AEl/FEDER, UE) grants RTI2018-101427-B-I00 to OP and RTI2018-094414-A-I00 to AE.

## Conflict of Interest

The authors declare that this manuscript has been prepared in the absence of any commercial or financial relationships that could be construed as a potential conflict of interest.
